# Supplementary material for: Alleviation of Memory Deficit by Bergenin via the Regulation of Reelin and Nrf-2/NF-κB Pathway in Transgenic Mouse Model
Source: Int J Mol Sci. 2021 Jun 20;22(12):6603. doi: 10.3390/ijms22126603 (PMC8234641; doi:10.3390/ijms22126603)
Supplement: Supplementary file 1 [file ijms-22-06603-s001.zip › ijms-1229788-supplementary.pdf]

International Journal of *Molecular Sciences*

MDPI

Supplementary

Alleviation of Memory Deficit by Bergenin via Regulation of Reelin and Nrf-2/NF-κB Pathway in Transgenic Mouse Model

Bushra Shal [1], Adnan Khan [1], Ashraf Ullah Khan [1], Rahim Ullah [2], Gowhar Ali [2], Salman ul Islam [3], Ihsan ul Haq [1], Eun Kyoung Seo [4,*] and Salman Khan [1,*]

[1] Pharmacological Sciences Research Lab, Department of Pharmacy, Faculty of Biological Sciences, Quaid-i-Azam University, Islamabad, Pakistan; bushra.shal@gmail.com (B.S.); adkhan165sbbu@gmail.com (A.K.); ashrafwazir6@gmail.com (A.U.K.); ihsn99@yahoo.com (I.H.)
[2] Department of Pharmacy, University of Peshawar, Peshawar, Pakistan; rphrahimullah@gmail.com (R.U.); gowhar_ali@uop.edu.pk (G.A.)
[3] School of Life Sciences, College of Natural Sciences, Kyungpook National University, Daegu, South Korea; dr_ssulman@yahoo.com
[4] College of Pharmacy, Graduate School of Pharmaceutical Sciences, Ewha Womans University, Seoul 03760, South Korea
* Correspondence: yuny@ewha.ac.kr (E.K.S.); skhan@qau.edu.pk (S.K.); Tel.: +82-2-3277-3047

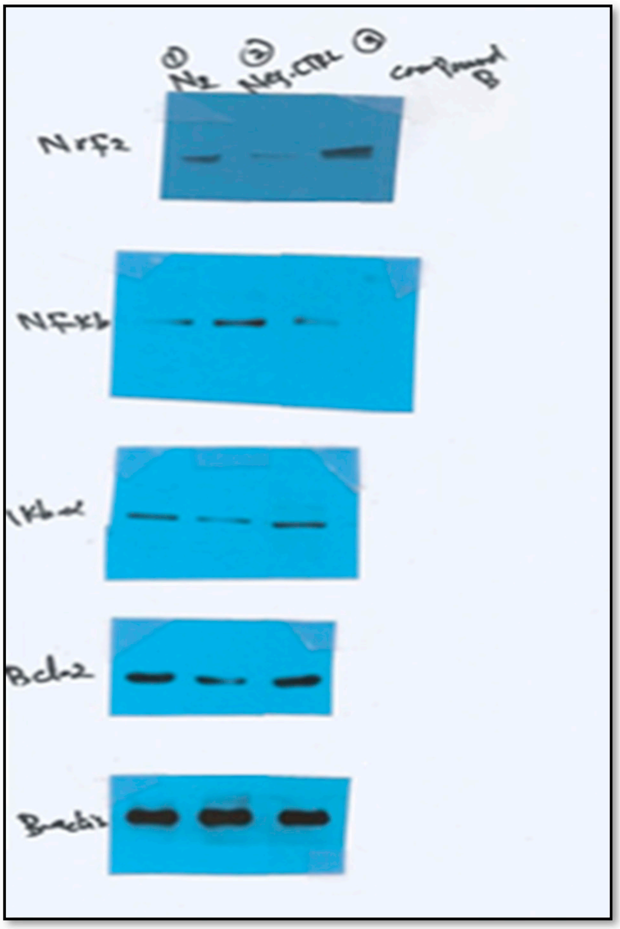

Figure S1. Uncropped full-length images of western blot membranes for the analysis of Nrf-2, NF-κB, IκB-α, and Bcl-2 proteins in hippocampal region of the wild type (N_2), 5xFAD Tg (Neg-CTRL) and Bergenin (Compound B) treated groups. Representing the western blot analysis shown in Figure 7.
